# Laparoscopic Surgery for Ovarian Cyst Infection with Avoidance of Ureteral Injury and Uterine Perforation following Intrauterine Insemination after Abdominal Modified Radical Trachelectomy

**DOI:** 10.1155/2019/8607417

**Published:** 2019-04-30

**Authors:** Moito Iijima, Shigenori Hayashi, Yusuke Kobayashi, Kosuke Tsuji, Eiichiro Tominaga, Kouji Banno, Daisuke Aoki

**Affiliations:** Department of Obstetrics and Gynecology, Keio University School of Medicine, Tokyo 160-8582, Japan

## Abstract

Pelvic inflammatory disease (PID) sometimes develops after intrauterine insemination (IUI). We herein present a case of PID which developed after IUI performed after abdominal modified radical trachelectomy (AmRT) and was treated with laparoscopic surgery. To our knowledge, this is the first case report of laparoscopic surgery for PID that occurred after AmRT in Japan. A 39-year-old woman who was diagnosed with cervical cancer stage IA1 with lymphovascular invasion underwent AmRT and pelvic lymphadenectomy. At 3 years and 6 months after the surgery, she had fever and pain in her left lower abdomen 10 days after IUI. She was diagnosed with PID with left ovarian cyst infection and underwent laparoscopic left ovarian cystectomy. Before surgery, bilateral ureteral catheters were inserted because of possible difficulty identifying the ureters. During surgery, severe adhesion was seen in the pelvic cavity. By moving the catheters manually back and forth from outside the body, we were able to identify the ureters visually. A uterine manipulator was inserted during surgery, rather than before surgery, to avoid the risk of uterine perforation. Laparoscopic surgery with ureteral catheters and a uterine manipulator can be applied safely for such cases after AmRT even when severe intraperitoneal adhesion is present.

## 1. Introduction

Cervical cancer is the fourth most common cancer in women worldwide [1]. Because this cancer often affects women of childbearing age (19-45 years), fertility-sparing surgery is an important issue [2]. Abdominal radical trachelectomy (ART) is a fertility-preserving surgery for young women with early-stage cervical cancer who want to have children. After this surgery, infertility treatment such as intrauterine insemination (IUI) and in vitro fertilization-embryo transfer (IVF-ET) is often required for pregnancy [3]. There are several reports on development of pelvic inflammatory disease (PID) after IVF-ET or IUI [4–6].

Ureteral injury is one of the most serious complications of gynecologic surgery. Prolonged postoperative morbidity leading to fistula formation, sepsis, or renal functional loss can occur after unrecognized ureteral injury [7, 8]. Adhesion, which is frequently formed in patients with a history of operations or inflammatory peritoneal processes, is significant risk factor of urologic injury during gynecologic surgery [9, 10]. According to the literature, ureteral stents are helpful when standard attempts to identify the ureter had failed in an area of severe adhesion [11].

Here, we report a case treated with laparoscopic surgery for PID following IUI after abdominal modified radical trachelectomy (AmRT), and we discuss some key points for safe performance in this surgery, especially for avoidance of ureteral injury. To our knowledge, this is the first case report of laparoscopic surgery for PID following AmRT in Japan.

## 2. Case Presentation

A 39-year-old woman, gravida 1, para 0, was diagnosed with invasive squamous cell carcinoma of the cervix following conization. Pathological findings showed carcinoma consistent with FIGO stage IA1 with lymphovascular invasion. She was referred to Keio University Hospital. The patient and her husband were informed of the treatment options, including AmRT and pelvic lymphadenectomy. The patient was told that the outcome of this procedure could not be guaranteed because an insufficient number of these procedures have been performed worldwide to yield reliable conclusions. The patient wished to preserve fertility, and she and her husband signed a written consent form agreeing to this treatment. Pathological findings after AmRT and pelvic lymphadenectomy showed no residual tumor and no lymph node metastasis. There was no finding of an ovarian tumor before surgery. A left ovarian cyst of 4 cm was identified during postoperative follow-up.

**Figure 1:**
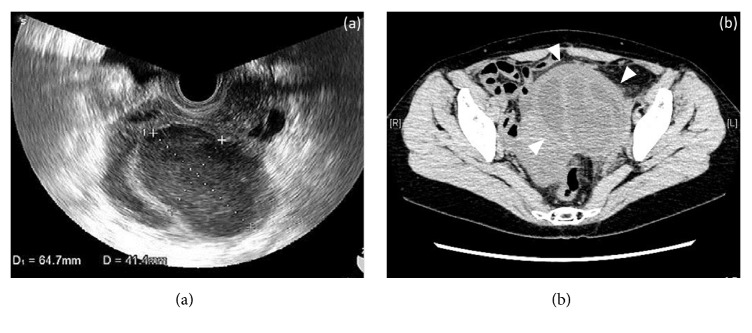
(a) Transvaginal ultrasonography at the first visit of the patient, showing a cystic tumor in the left adnexal area. (b) Pelvic CT on day 8 of hospitalization, showing that the infectious left ovarian cyst had grown to 10 cm (arrowheads).

At 3 years and 6 months after surgery, the patient underwent IUI and then had fever and pain in her left lower abdomen 10 days later. At her first visit, her temperature was mildly elevated to 37.5°C. The patient's pregnancy was denied because a qualitative urine human chorionic gonadotropin (hCG) test was negative. A tumor with tenderness was palpated in the left adnexal area. A cystic tumor of 64x 41 mm was found by transvaginal ultrasonography (Figure 1(a)). Blood tests showed increases in white blood cell (WBC) count to 11900/*μ*L and C-reactive protein (CRP) to 22.80 mg/dL. The patient was diagnosed with PID with ovarian cyst infection and hospitalized for treatment. Conservative treatment with antibiotics was initially used, but her symptoms did not improve. On hospital day 8, blood tests showed a further increase in WBC count to 23900/*μ*L and CRP to 28.17 mg/dL, and pelvic CT showed that the ovarian cyst had grown to 10 cm in size (Figure 1(b)).

**Figure 2:**
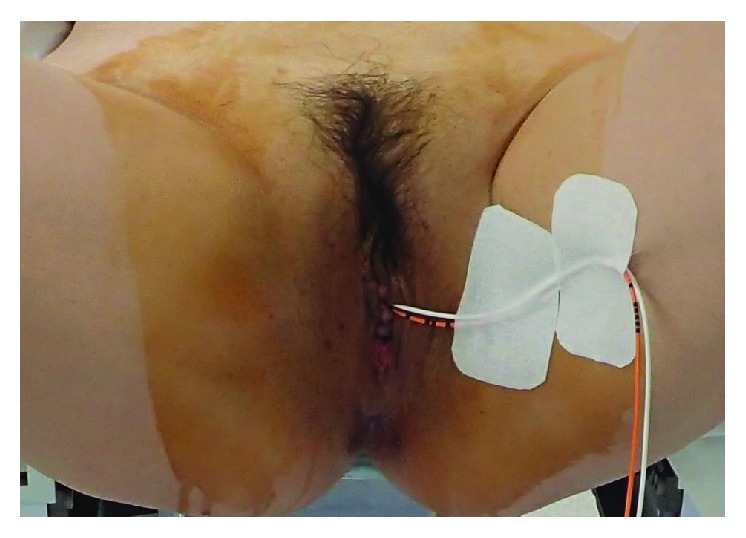
Fixation of the ureteral catheters to the thigh with tape. In this case, the left and right sides of the ureter where the catheter was inserted were discriminated by color. The white catheter was inserted into the right ureter, and the orange one was inserted into the left ureter.

We decided to perform laparoscopic left ovarian cystectomy on day 8. Since the patient had a history of open surgery, adhesion was likely in the abdominal cavity. Before surgery, we asked the urologist to insert bilateral 6 Fr ureteral catheters because of possible difficulty identifying the ureters. The catheters were fixed to the thigh with tape (Figure 2). The uterine manipulator was not inserted before surgery to avoid the risk of uterine perforation.

**Figure 3:**
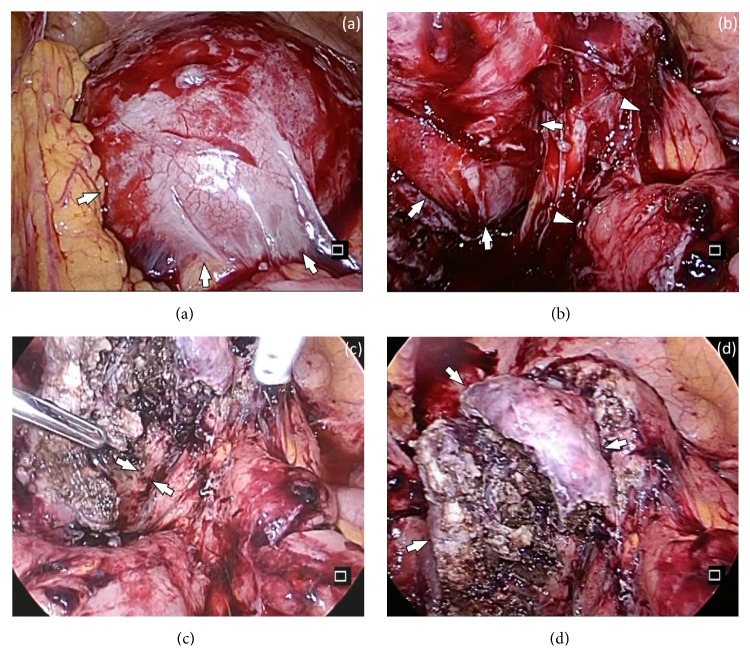
Laparoscopic views during the surgery. (a) The left ovary was swollen to 10 cm (arrows). (b) The uterine was firmly adhered with the left adnexa (arrows) and retroperitoneum (arrowheads). (c) The left ureter (arrows) was identified visually by moving the catheter manually back and forth from outside the body. (d) The left ovarian cyst was excised, leaving the normal part of the ovary (arrows).

CO_2_ pneumoperitoneum was established at 10 mmHg. Laparoscopic left ovarian cystectomy was performed using typical trocar placement. The left ovary was swollen to 10 cm and the fluid contents were purulent (Figure 3(a)). The left adnexa, posterior uterine wall, and retroperitoneum were firmly adhered (Figure 3(b)). The bilateral fallopian tubes were firmly adhered to the surrounding tissue and were unable to be identified. During surgery, by moving the catheter manually back and forth from outside the body, we were able to identify the ureters visually (Figure 3(c)). The 7 cm uterine manipulator was inserted during surgery under a laparoscopic view. The left ovarian cyst was excised, leaving the normal part of the ovary (Figure 3(d)). The operation time was 2 h and 58 min, and blood loss was 550 mL. No complications occurred during or after surgery. After the operation, symptoms improved rapidly and the patient was discharged 8 days after surgery. The excised specimen was pathologically an endometriotic cyst. A bacterial culture of the cyst fluid was positive for Prevotella bivia, Prevotella species, and Finegoldia magna.

## 3. Discussion

In this case, there were two main points in the surgery. First, before surgery we asked the urologist to insert bilateral ureteral catheters to permit identification of the ureters, since the patient had previously undergone AmRT and pelvic lymphadenectomy. During surgery, by moving the catheter manually back and forth from outside the body, visual identification of each ureter was possible. Using this method, we reduced the risk of ureter injury. Second, the uterine manipulator was not inserted before the operation, but inserted under a laparoscopic view during surgery to avoid the risk of uterine perforation. Appropriate manipulation secured the field of view and provided an understanding of the anatomy. A high level of difficulty is likely in laparoscopic surgery in cases with extensive adhesion, especially after malignant tumor surgery such as radical trachelectomy with pelvic lymphadenectomy, and the preoperative preparations in this case were important.

Vaginal or abdominal radical trachelectomy is currently feasible for treatment of patients with early-stage cervical cancer as fertility-sparing surgery in gynecological oncology [12, 13]. After this surgery, infertility treatment is usually necessary to achieve conception [3, 14, 15]. For example, Kasuga et al. showed that, among patients who gave birth after 22 weeks of pregnancy after ART, 67% (22/33) needed infertility treatment such as IUI or IVF-ET [3]. The incidence of infection after oocyte retrieval is about 0.4% [16, 17]. Since PID is usually due to ascending infection from the cervix, IUI and embryo transfer can also theoretically place the patient at increased risk for this complication [5]. Patients who undergo radical trachelectomy have a high rate of infertility treatment, and this increases the risk of infection. PID in women with endometriosis is severe and refractory to antibiotic treatment and often requires surgical intervention [18]. For example, Elizur et al. showed that, among patients hospitalized with PID or tubo-ovarian abscess in a tertiary referral center, those with endometriosis were significantly more likely to have undergone a fertility procedure compared with those without endometriosis, and more frequently experienced a severe and complicated course involving longer hospitalization and antibiotic treatment failure [18].

In this case, we performed laparoscopic surgery for PID after AmRT. Laparoscopic surgery with ureteral catheters and a uterine manipulator can be applied safely for such cases after AmRT, even if severe intraperitoneal adhesion is present. There are several key points for safe performance of this surgery: the uterine manipulator should be inserted under a laparoscopic view to avoid uterine perforation; manipulation of this instrument secures the field of view and facilitates anatomical understanding; by inserting of ureteral catheters and moving the catheters manually from outside the body, visual identification of the ureters is possible and the risk of ureter injury can be reduced. Patients who have undergone radical trachelectomy often require infertility treatment such as IUI or IVF-ET, which increases the risk of infection. Therefore, clinicians should be alert to potential infectious morbidity following such treatment and should recognize the importance of early diagnosis and intervention for minimizing the morbidity.
